# Concordance between noninvasive assessments of fibrosis in patients with HIV/HIV+HBV infection

**DOI:** 10.1186/1471-2334-14-S4-O19

**Published:** 2014-05-29

**Authors:** Elena Dumea, Adrian Streinu-Cercel, Sorin Rugină, Lucian Cristian Petcu, Simona Claudia Cambrea

**Affiliations:** 1 Ovidius University, Constanța, Romania; 2 Clinical Hospital of Infectious Diseases, Constanța, Romania; 3 Carol Davila University of Medicine and Pharmacy, Bucharest, Romania

## 

Many studies used non-invasive methods to estimate the prevalence of significant fibrosis and its risk factors in patients with HIV infection. We evaluated the ability of APRI and FIB-4 score to differentiate between the different stages of fibrosis (no fibrosis/minimal fibrosis = F0-F1 and F2-F4 fibrosis moderate-severe/cirrhosis), taking as a reference, in the absence of liver biopsy, the hepatic fibrosis stratification by FibroScan.

Group 1 was represented by 39 patients with HIV infection and group 2 by 71 patients with HIV/HBV coinfection. AUROC was used to calculate for each group and for each score the optimal value for identifying significant fibrosis. Then we determined the cut-off value that identifies significant fibrosis with maximum specificity. The Kappa score was then calculated for the concordance between methods.

For HIV/HBV coinfected patients, to identify significant fibrosis score on tally Kappa classification for fibrosis by APRI versus FibroScan, Kappa=0.494, 95% CI (0.245, 0.742) on the identification of fibrosis (F0-F1 to F ≥2), for the FIB-4 Kappa=0.481, 95% CI (0.238, 0.725) for both the moderate concordance. Regarding the comparison of the two methods APRI and FIB-4 kappa=0.698, 95% CI (0.485, 0.910), significant concordance.

For patients with HIV to identify significant fibrosis Kappa score tally on the classification of fibrosis by APRI versus FibroScan Kappa=0.217, 95% CI (-0.424, 0.858) on the identification of fibrosis (F0-F1 to F ≥2), for the FIB-4 Kappa=0.164, 95% CI (-0.451, 0.779) for both the correlation is reduced. Regarding the comparison of the two methods APRI and FIB-4 kappa=0.217, 95% CI (-0.424, 0.858), which confirms the low correlation.

There is sufficient evidence that the tests used: APRI and FIB-4 have the ability to distinguish for both groups of patients between the two classes of fibrosis (F0-F1 to F≥2) meaning between patients with and without liver disease.

Although for patients with HIV infection a low concordance was noted between non-invasive methods for the diagnosis of fibrosis, in coinfected patients it was moderate and these tests could be used as evaluation methods in the monitoring of liver injury especially when the results of these tests are concordant.

